# Setting Regional Priorities for Palliative and End-of-Life Care Research Using a Delphi Technique Approach

**DOI:** 10.1177/08258597241264455

**Published:** 2024-07-26

**Authors:** Nikolaos Efstathiou, Ping Guo, Wendy Walker, John I. MacArtney, Cara Bailey

**Affiliations:** 1 1724 University of Birmingham, College of Medical and Dental Sciences, Institute of Clinical Sciences, School of Nursing and Midwifery, Birmingham, UK; 2 School of Nursing, University of Ottawa, Ottawa, Canada; 3 The Royal Wolverhampton NHS Trust, Wolverhampton, UK; 4 Unit of Academic Primary Care, 2707 University of Warwick, Warwick, UK

**Keywords:** Delphi technique, research priorities, palliative care, end-of-life care

## Abstract

**Objective:**

Identifying research priorities is very important for palliative and end-of-life care to ensure research is focused on evidence gaps. This project aimed to identify and prioritise palliative and end-of-life care research areas within the West Midlands region in United Kingdom (UK).

**Methods:**

A modified Delphi technique approach was used with palliative care stakeholders. The first round was item generation via rapid interviews. Data were analysed using content analysis and all the items were grouped into main categories. For round two, an online survey was conducted to present all the items from round one, and stakeholders were asked to rate the priority of items on a Likert-type scale (1 = not a priority to 7 = essential priority). Items that achieved consensus in round two were presented to the third round, where stakeholders ranked them in descending order.

**Results:**

We completed and analysed 56 rapid interviews which resulted in 158 research items under 15 categories. The research items were rated by 30 stakeholders and seven items which reached consensus were subsequently ranked in order by 45 stakeholders. The highest ranked item was ‘Integrated care systems to prevent crisis’, followed by three research items related to ‘equity’ in palliative care.

**Conclusions:**

Our research priorities, although unique for our region, mirror previously research priorities from other regions and countries. This suggests issues of integration and equity in palliative and end-of-life care remain unresolved, despite ongoing initiatives and research to address these issues.

## Introduction

Palliative care is a holistic approach to relieving health-related suffering for people living and dying.^1^ It is required by an estimated 56.8 million people each year worldwide, however only around 14% of those in need currently receive it.^2^ Palliative care demands are expected to increase substantially in the future, with moderate increase estimates of 25% (from 375 398 to approximately 470 000 people per year) by 2040 in England and Wales alone.^3^ Globally healthcare organisations have identified the need for integrating palliative care in the disease trajectory of people with chronic and life-threatening conditions and most countries have responded by updating or developing national policies to address the increasing demand for palliative care.^4^ In England, statutory guidance specifies a duty to commission palliative care services in the context of end-of-life care.^5^ However, as more people approach the end of life with advanced age-related ill-health,^6^ and societal preference for a non-institutionalised death grows,^7^ so too does the need for investment in palliative and end-of-life care research for service improvement to be realised.

Palliative care, like any other specialised service, should be based on robust evidence. Despite a fourfold increase of published research in palliative care from 2002 to 2020 and a further estimated 19% increase by 2025 as identified in a bibliographic review, there are still concerns that most studies are related to cancer and there is limited cooperation between researchers from different fields.^8^ The authors of this bibliographic review suggested that setting palliative care research priorities may expand the focus of palliative care beyond cancer and enhance collaboration between researchers.^8^

Identifying research priorities is very important in any discipline to ensure research is focused on evidence gaps, and even more so for palliative and end-of-life care where the proportion of research funding is smaller than other disciplines.^9^ An international systematic review identified 10 studies that had produced palliative care research priorities.^10^ The overall findings of the systematic review included palliative care research priorities in the areas of communication, inequalities, continuity of care, well-being, recognising family needs, and training.^10^ In terms of the UK-based studies included in this review, the priorities setting had focused on end-of-life care,^11^ palliative care,^12^ generalist end-of-life care,^13^ and palliative and end-of-life care in the emergency setting,^14^ and most studies were more than 10 years old. In addition, the James Lind Alliance and the Palliative and End of Life Care Priority Setting Partnership (PeolcPSP) sought to determine the research priorities that UK patients, carers, and health and social care professionals involved in end-of-life care believed were most important.^15^ The top research priority was related to ways of preventing palliative care crises that would help patients to stay in their place of choice.^15^ While in the years that followed the work of the PeolcPSP, most of the identified research priorities attracted some research interest and funding, it has been suggested that there is still a need for further research to address the issue of palliative care crises ‘out of hours’.^16^

In the West Midlands, UK, a commitment to improving palliative care has been strengthened through the development of a National Institute for Health and Care Research (NIHR) funded palliative care research hub (https://www.birmingham.ac.uk/schools/nursing/research/brhumb/index.aspx). The research hub aims to develop palliative care evidence aligned to the priorities of the regional communities and be responsive to local challenges and needs. Involving regional stakeholders was imperative since evidence suggests that stakeholder involvement reduces “research waste”, promotes the relevance and legitimacy of research, and increases the uptake of research findings.^17^ An important programme of work within the first year of inception, and the aim of the reported project here, was to identify stakeholder views on priority areas for palliative and end-of-life care research that would serve as a basis for impactful future research.

## Methods

### Study Design

A three-round modified Delphi technique approach was used to identify palliative and end-of-life care research priorities within the West Midlands region in UK between April 2022 and November 2022. Consensus research methods, such as Delphi approaches have been used widely in healthcare research and more specifically in identifying research priorities.^18,19^ The Recommendations for the Conducting and REporting of DElphi Studies were used as a guidance to report this project.^20^

### Setting and Participants

This project took place at West Midlands, UK. The population in this region of UK is ethnically diverse and, due to high levels of socioeconomic deprivation, experience above national average rates of premature deaths from preventable conditions.^21^ Although the proportion of deaths in hospitals has decreased in the region, hospitals remain the most common place of death. In addition, regional data shows high rates of emergency admissions at the last 12 months of life, with patients spending more than six weeks in an acute hospital bed the last year of their lives. There are more than 20 hospices in the region for adults and children, and specialist palliative care teams provide community services. Ratings for end-of-life care by the independent regulator of health and adult social care in England are generally good for most settings.^22^

Participants in this project were healthcare professionals across health and social care settings and services in the West Midlands region in UK where palliative and end-of-life care is provided. In addition, lay people who had experienced palliative or end-of-life care participated in this project.

### Data Collection and Analysis

#### Round 1

The first round of the modified Delphi aimed to generate items (perceived palliative and end-of-life care research priorities) to be considered in further rounds. Instead of the classical first round Delphi technique method of using a questionnaire to generate items,^23^ we conducted rapid qualitative interviews with stakeholders from the West Midlands region representing professional and lay perspectives. Rapid qualitative interviews gained popularity during the COVID-19 pandemic to produce timely evidence,^24^ and fitted well with our aim to produce items for the subsequent rounds to inform research activities in the near future.

A purposive sampling strategy was adopted, and we recruited (n = 49) medical, nursing, paramedical healthcare professionals, and social workers, representing both generalists and specialists in palliative and end-of-life care, providing services in hospitals, community settings, and the third sector (hospices, palliative and end-of-life charities) within the UK's West Midlands region. Furthermore, we recruited lay stakeholders (n = 7), ‘experts by experience’, members of the patient and public involvement group of our West Midlands regional research hub.

An interview guide comprising four questions (Supplementary file 1) was used to explore stakeholders’ views on local priorities for palliative and end-of-life care research. Rapid interviews were conducted by the project team remotely, either via telephone, or an on-line virtual meeting. Each interviewer kept notes to document and collate views during telephone interviews, or used the transcription function of the virtual meeting platform to record participants’ views, as is common in rapid qualitative interviews.^24^

The data collected in round one, was analysed by one researcher (NE) using a content analysis approach.^25^ The aim was to create categories under which to represent the research items, hence we focused on manifest analysis, where the analysis stays at the ‘surface’, describing what the participant actually said, and the visible and obvious in the interview data.^26^ The outcome of content analysis was made available to the co-research team (CB, JM, PG, WW) who subsequently met to discuss and finalise the structure of the round two survey, and to confirm the items within each category.

#### Round 2

For the second round we developed an online survey based on items generated in round one. The survey was developed and administered via *online surveys* run by Jisc^©^. The same stakeholders from round one were sent an email with information about the study, the link to the survey, and instructions on how to complete it. Stakeholders were asked to rate the extent to which they agreed or disagreed on a seven-point Likert scale (1 = not a priority to 7 = essential priority) for each of the research items in the survey. We also shared the inclusion criteria and encouraged them to share the link of the survey with other stakeholders within the region. Round two was open from 15 August- 23 September 2022. Reminders were sent three weeks after the survey went live.

Descriptive statistics were used in round two [median and interquartile range (IQR)] for each item in the survey, and consensus was a priori set at ≥80% of participants rating an item as 6 = high priority and 7 = essential priority.

#### Round Three

In the third round, only items that met consensus criteria were included, and stakeholders from round one were sent an invitation via email to access the survey, which was asking to rank in order these items in descending order, from one to seven. Within the survey instructions, we asked stakeholders to suggest more specific research questions for their highest ranked area of research. We asked them again to share the link of the survey with other stakeholders within the region. Round three was open from 1 October – 10 November 2022. Reminders were sent again three weeks after the survey went live.

For the third round, the median and IQR scores were used to develop a list of priorities in descending order. A lower median score in round three would indicate highest priority, and the lower the IQR would show less dispersion of rankings around the median, indicating agreement among the participants.^27,28^

### Ethical Considerations

Project governance was assessed and assured by the host organisation. The project was a stakeholder engagement and consultation activity, and hence research ethics approval was not required (The University of Birmingham Research Governance and Integrity Committee waived the requirement for ethical approval). No personal or sensitive data were collected, and the principles of anonymity and confidentiality were upheld throughout.

## Results

For the first round we completed 56 rapid interviews which lasted on average 15 min. Following content analysis, we created 15 categories which represented the 158 areas of palliative and end-of-life care research identified by the stakeholder participants (Table 1). The category ‘Equity’ included the highest number of items, however there were some overlaps between items within this category.

**Table 1.** Categories and Number of Items.

Categories	No of items
Equity	19
Symptom Control, Medications Management/Alternative Therapies	15
Service Development and Evaluation	15
Models of Care	15
Challenging and Complex Issues	14
Bereavement	13
Training	11
Integrated Care and Joint Working	10
Person Centred Care	9
Patient and Family Needs	8
Community Care and Engagement	7
Workforce Issues	6
Prognosis	6
Communication	6
Advance Care Planning	4

Thirty stakeholders responded to round two. Twenty (66.7%) of the 30 stakeholders had participated in round one. The items with the highest median score and lower IQR for each of the categories is presented in Table 2. Only a few items attracted lower ratings by the stakeholders (no priority = 19; low priority = 57). Among the lowest prioritised items were the ‘Use of creative arts to facilitate advance care planning’ that was rated as no or low priority by 30% (n = 9) of the stakeholders, while six participants (20%) rated ‘Historical grief and pandemic - impact on LGBTQ + community in relation to AIDS’ as a low or no priority. Although several items were scored high, there was wide dispersion of scoring resulting in only seven items meeting the consensus criteria, which were then included in round three.

**Table 2.** Highest Rated Item Within Each Category.^a^

Categories	Items	Median (IQR)	No (%) of participants rating as 6 = high priority and 7 = essential priority
Advance Care Planning	The blockages to earlier advance care planning conversations and how they can be facilitated	6 (1)	23 (76.7%)
Bereavement	Bereavement support for the wider family eg siblings	6 (1)	19 (63.3%)
Communication	Barriers in communicating end-of-life	6 (.75)	25 (83.3%)
Community Care and Engagement	Alternative ways to support more patients and families, enabling patients and families to receive support in their community	6 (.75)	22 (73.3%)
Challenging and Complex Issues	Continuity of care, including transition between settings and communication between care teams	6 (1)	23 (76.7%)
Equity	Address inequality for underrepresented populations, eg, old people and ethnic minority groups	6 (.75)	24 (80.0%)
Integrated Care and Joint Working	Integrated care systems to prevent crisis	6 (1)	24 (80.0%)
Models of Care	Quality of end-of-life care in care homes	6 (1.25)	20 (66.7%)
Patient and Family Needs	Community care meeting the needs of ethnic minority groups	6 (1.75)	18 (60.0%)
Person Centred Care	To enable patients to live well as long as possible and to have the end-of-life experience that they would like	6 (2)	19 (63.3%)
Prognosis	Recognising end-of-life and forward planning	6 (.75)	23 (76.7%)
Service Development and Evaluation	What patients value in a service?	6 (2)	20 (66.7%)
Symptom Control, Medications Management/ Alternative Therapies	Thinking about access to medicines for symptom control in the community. This would encompass both discharge and access at short notice, in its widest sense e.g prescribing, medicines access and administration for prn.	6 (1)	19 (63.3%)
Training	How to upskill healthcare professionals to provide basic palliative care and know who to refer to specialist palliative care services	6 (1)	24 (80.0%)
Workforce Issues	Workforce well-being and resilience	6 (2)	20 (66.7%)

a Where median and IQR scores were the same for items, the item with the highest number of participants rated as essential priority was included.

Forty-five stakeholders responded in round three, who ranked in order the seven research priorities. Twenty (69%) of the 45 respondents had participated in round two. The item ‘Barriers in communicating end-of-life’ was ranked first by 13 (28.9%) stakeholders, however the IQR = 5 for this item demonstrated lack of agreement. Hence, we reviewed the rankings for each item to identify which item was ranked highest among the seven items with small spread of rankings, using a low median and IQR scores. The item of ‘Integrated care systems to prevent crisis’ was ranked highest by most respondents with low IQR score (median = 3, IQR = 2), demonstrating agreement among the stakeholders (Table 3).

**Table 3.** Top Research Priorities in Descending Order.

Research priority item	Median (IQR)	No (%) of participants ranked item as 1 (highest priority)
1. Integrated care systems to prevent crisis	3 (2)	9 (20%)
2. How to make palliative care services more accessible/relevant to groups currently underrepresented in our patient cohort (non-malignant disease, ethnic minorities, those with a learning disability, LGBTQ+, prisoners etc)	3 (3)	9 (20%)
3. Address inequality for underrepresented populations, eg, old people and ethnic minority groups	4 (2)	3 (6.7%)
4. Inequalities and access to palliative care services by marginalised groups	4 (3)	8 (17.8%)
5. Barriers in communicating end-of-life	4 (5)	13 (28.9%)
6. Palliative care and social care - lack of social care	5 (3)	2 (4.4)
7.How to upskill healthcare professionals to provide basic palliative care and know who to refer to specialist palliative care services	5 (4)	8 (17.8%)

Six out of the nine stakeholders who ranked ‘Integrated care systems to prevent crisis’ as their top priority suggested more focused research questions, of which some were very similar. Here we report an edited version of these research questions: Is advance care planning effective in preventing crises? What are the essential elements of integrated health and social care in end-of-life care that can prevent crises? What is the effectiveness of integrated care in preventing or managing crises? How can we improve communication between services and systems?

## Discussion

A three round modified Delphi approach was used to develop a list of palliative and end-of-life care research priorities within the West Midlands region of UK to allow a newly developed research hub to focus on research that would be of value to the local community. Following an iterative approach, the highest ranked item was ‘Integrated care systems to prevent crisis’, with some additional suggestions from the participants to focus further our efforts in this research area.

Preventing palliative and end-of-life crises has been a long-standing research priority. It was also identified as a top research priority in the 2015 PeolcPSP priority setting study.^15^ This priority is of particular importance, considering the regional burden of emergency admissions at the end-of-life which are usually due to palliative and end-of-life care crises.^22^ A combination of ageing, multi-morbidity and increased fluctuations in the end-of-life trajectory means that the end of life is more complex, uncertain and there is an increased risk of triggering a palliative care crisis.^29^ More specifically, in our project participants emphasised the ‘integration of care systems’ in preventing crises; an important national policy initiative and driving force behind personalised and responsive needs-based care.^30^ Modern healthcare seeks an integrated approach, where patients’ and their families’ needs are considered by different disciplines,^31^ however it is often a challenge to achieve.^32,33^

A particular area of research that was highly rated by the participants was related to equity in palliative care, with three items under the category ‘Equity’ achieving consensus in round two, and ranked second, third and fourth in the third round. Factors such as education, social isolation, income, housing, and disability are widely seen as determinants of healthcare inequalities,^34^ and could equally affect inequality in palliative and end-of-life care. This is of particular concern in the West Midlands region where employment rates are lower, and people have shorter life expectancy than other regions in England and on average spend 20 years of their lives in poor health.^21^ Several studies globally have also identified racial and ethnic inequities in access to palliative and end-of-life care and specialised facilities where it is offered, such as hospices.^35,36^ The West Midlands region has a diverse population with more than 23% being from a non-white ethnic group and specific areas such as Birmingham having 51% of the population Black, Asian or other minority ethnic groups.^37^ In addition, attention is required in equitable access to palliative care since cancer tends to be the most common condition for which patients receive palliative care both locally and globally.^34,38^ Equity in palliative care has been constantly identified as one of the research priorities in priority setting studies as reported by Hasson et al in their systematic review of international palliative care research priorities.^10^ Considering the ethnic diversity of the West Midlands region and the existing factors that lead to healthcare inequalities, it was not surprising that this area was identified as a high research priority by our project's stakeholders.

The fifth research priority suggested research around the ‘Barriers in communicating end of life’. The advantages of having early conversations with patients and their families about end-of-life care are well established, however clinicians experience obstacles when having conversations about end-of-life care, such as prognostic uncertainty, concerns about upsetting patients, navigating patient readiness, and feeling unprepared for these conversations.^39^ These obstacles and their impact may be intensified within a region of high levels of ethnic minorities, if there is lack of cultural and religious understanding, limited access to translators and low availability of training for healthcare professionals. A recent modified Delphi study in Australia also identified *improving communication about prognosis* among the 10 top research priorities,^40^ demonstrating the global need for research in the barriers of communicating end of life.

Under the category of ‘Integrated Care and Joint Working’, the item *‘*Palliative care and social care - lack of social care’ was ranked sixth in round three. This item indicated a perceived situation (lack of social care) and an acknowledgement that social care is an important element of palliative care. Indeed, the NHS Confederation has reported that social care in England, Wales and Northern Ireland is underinvested and challenged by difficulties to find and retain employees, resulting in seeing fewer patients, who are then more dependent on healthcare services, less independent, and more vulnerable.^41^ Further exploration would be required to identify more focused research questions that could explore issues under this item.

Although ranked last among the seven research priorities, ‘Upskilling generalists in basic palliative care skills’ appears to be an important issue in the UK, as UK ‘generalist’ numbers caring for patients at the end of life total around three million, and these healthcare professionals will provide most patient and family care across any setting in the final year of life.^42^ Further research would be required to identify regional generalists’ educational needs in palliative care skills and explore strategies to develop and maintain skills and expertise.

Following the completion of the palliative and end-of-life research priorities setting, we held an online regional collaboration event which enabled health and social care professionals, the research team and an ‘expert by experience’ group to collaboratively develop the parameters of a grant on integrated palliative care to prevent crises. Findings of the priority setting were presented, and participants worked in smaller groups facilitated by members of the project (CB, JM, NE, PG) to define integrated care, models of good practice, areas of challenge, and develop specific research questions and potential methods of enquiry. The collaboration event inspired three potential research studies and grant funding applications designed to address the priorities for palliative and end-of-life care research reported in this paper.

### Limitations

This was a regional project to identify palliative and end-of-life care research priorities. Despite the representation of different stakeholders, we acknowledge that findings cannot be generalised. However, transferability may be possible in similar contexts. Providing complete anonymity in the survey rounds, meant that we could not collect and present any demographic information about the participants; however, our engagement philosophy fostered a commitment to represent a variety of stakeholders’ viewpoints in every round of the Delphi study. Participants identified topics for palliative and end-of-life care research, rather than focused research questions. This is quite common in studies that aim to set research priorities.^18^ We tried to elicit more focused research questions in round three, and we held an online collaboration event to refine research questions. Finally, having three items related to equity in round three may have contributed to dispersion of rankings for each of these items resulting in them ranked second to fourth.

## Conclusions

Setting priorities for research is useful to help increase multidisciplinary collaboration and ensure researchers are focused on investigating topics important to their communities. We identified seven palliative and end-of-life care research priorities that will guide future research in our region. Our research priorities mirror previously reported research priorities from other regions and countries, as well as echoing international policies advocating for integrated palliative care and prevention of crises to allow people to receive care and die at their preferred place. While the findings are not dissimilar to what has already been reported in the literature or culturally similar countries, similarity served to strengthen the regional priorities for palliative and end-of-life care research. We also anticipate our methods may be helpful to other researchers and clinical groups embarking on regional research priority setting. Although there has been some research into each of the identified priority areas, their continued appearance in priority setting studies suggests that these issues persist in crucial areas of palliative and end-of-life care. In particular, practice-focused knowledge and understanding of how crisis can be averted and how international models of working can be applied and contextualised to support local service improvement require significant attention through investment in further research.

## Supplemental Material

sj-docx-1-pal-10.1177_08258597241264455 - Supplemental material for Setting Regional Priorities for Palliative and End-of-Life Care Research Using a Delphi Technique Approach Supplemental material, sj-docx-1-pal-10.1177_08258597241264455 for Setting Regional Priorities for Palliative and End-of-Life Care Research Using a Delphi Technique Approach by Nikolaos Efstathiou, Ping Guo, Wendy Walker, John I. MacArtney and Cara Bailey in Journal of Palliative Care

sj-docx-2-pal-10.1177_08258597241264455 - Supplemental material for Setting Regional Priorities for Palliative and End-of-Life Care Research Using a Delphi Technique Approach Supplemental material, sj-docx-2-pal-10.1177_08258597241264455 for Setting Regional Priorities for Palliative and End-of-Life Care Research Using a Delphi Technique Approach by Nikolaos Efstathiou, Ping Guo, Wendy Walker, John I. MacArtney and Cara Bailey in Journal of Palliative Care

## Abbreviations

IQR: Interquartile Range
PeolcPSP: Palliative and End of Life Care Priority Setting Partnership
UK: United Kingdom
