# Supplementary material for: Setting Regional Priorities for Palliative and End-of-Life Care Research Using a Delphi Technique Approach
Source: J Palliat Care. 2024 Jul 26;40(3):258–65. doi: 10.1177/08258597241264455 (PMC12130606; doi:10.1177/08258597241264455)
Supplement: sj-docx-1-pal-10.1177_08258597241264455 - Supplemental material for Setting Regional Priorities for Palliative and End-of-Life Care Research Using a Delphi Technique Approach [file sj-docx-1-pal-10.1177_08258597241264455.docx]

Supplementary file 1

Rapid interview questions

1. What do you consider are the priorities for future research in palliative and end-of-life care?

2. What are your thoughts about the current provision of palliative and end-of-life care in the West Midlands?

3. What examples of good practice are you aware of in the West Midlands for palliative and end-of-life care provision?
